# Different effects of mechano- and metaboreflex on the central blood pressure waveform

**DOI:** 10.3389/fphys.2024.1489412

**Published:** 2025-01-07

**Authors:** Nobuhiro Nakamura, Peng Heng, Naoyuki Hayashi

**Affiliations:** Faculty of Sport Sciences, Waseda University, Tokorozawa, Japan

**Keywords:** muscle mechanoreflex, muscle metaboreflex, central blood pressure, static passive stretching, augmentation pressure

## Abstract

**Introduction:**

The effect of mechanoreflex on central blood pressure (BP) is unclear, although the influence of metaboreflex has been investigated. A relatively small contribution of the mechanoreflex to the pressor response to exercise has been considered in humans because many studies have failed to isolate the mechanoreflex-mediated pressor response. In a recent study, we successfully isolated a mechanoreflex-mediated pressor response using static passive stretching (SPS) in the forearm. Thus, it is possible to isolate the effect of the mechanoreflex on the central BP using this recently developed method. We investigated the effect of muscle mechanoreflex on central BP and compared the changes in the shape of the central BP waveform during mechanoreflex and metaboreflex.

**Methods:**

We measured 12 healthy males (age, 26 ± 2 years; height, 171.1 ± 5.2 cm; body mass, 63.3 ± 10.3 kg; body fat, 16.7% ± 3.9%; means ± standard deviation [SD]) in this study. All participants performed static passive stretching (SPS) of the forearm for 60 s to isolate the muscle mechanoreflex. They also performed 120 s of isometric handgrip (IHG) at 30% maximal voluntary contraction and underwent 180 s of post-exercise ischemia (PEI) to isolate the muscle metaboreflex. The carotid BP (cBP) waveform was obtained from the right common carotid artery as the central BP waveform. We evaluated the first systolic peak (P1) and second systolic peak (P2) from the cBP waveform.

**Results:**

SPS increased cBP with an increase in P1 (*p* < 0.05), whereas PEI increased cBP with an increase in P2 (*p* < 0.05). SPS did not alter augmentation pressure (AP) (*p* > 0.05), whereas PEI increased it (*p* < 0.05). The relative change from rest (Δ) in P1 during SPS was positively correlated with that in stroke volume (r = 0.68; *p* < 0.05), and the ΔAP during PEI was positively correlated with that in total peripheral resistance TPR (r = 0.61; *p* < 0.05).

**Conclusion:**

These results suggest different effects of mechano- and metaboreflex on the change in shape of the central BP waveform; mechanoreflex and metaboreflex deform P1 and P2, respectively.

## 1 Introduction

The central blood pressure (BP) waveform has two systolic peaks: the first systolic peak (P1), which is generated by cardiac ejection and propagation of pressure to the peripheral arteries; and the second systolic peak (P2), which is generated by propagation from the peripheral arteries to the heart (Casey et al., 2008). P1 and P2 are influenced by stroke volume and peripheral vasoconstriction, respectively (Nichols, 2005; Cecelja and Chowienczyk, 2012).

Central BP measured in the aorta and carotid artery is strongly associated with damage to the heart, kidneys, and brain (Roman et al., 2007; Kollias et al., 2016). Central BP response to static exercise can potentially capture left ventricular hypertrophy (Chirinos et al., 2010). Other study has also suggested that central BP response to static exercise is useful in evaluating cardiovascular risk (Tanaka et al., 2014). Despite the importance of central BP, limited attention has been paid to the mechanism of central BP response during exercise.

Pressor responses to exercise are influenced by the exercise pressor reflex (EPR), a feedback mechanism involving receptors in exercising skeletal muscles (Gallagher et al., 2006). EPR is induced by two stimuli: the mechanoreflex originating from physical changes of exercising muscles (Nóbrega et al., 1994; Gladwell and Coote, 2002; Vianna et al., 2010; Venturelli et al., 2019) and the metaboreflex originating from chemical changes in muscles (Scherrer et al., 1990; Ichinose et al., 2006; Crisafulli et al., 2015).

The effect of the mechanoreflex on central BP is unclear, whereas the influence of the metaboreflex has been investigated (Edwards et al., 2008; Kalfon et al., 2015; Figueroa et al., 2020). The metaboreflex increases aortic systolic BP depending on the reflected wave induced by the systole of the heart (Edwards et al., 2008; Kalfon et al., 2015; Figueroa et al., 2020). A relatively small contribution of the mechanoreflex to the pressor response to exercise has been considered in humans because many studies have failed to isolate the mechanoreflex-mediated pressor response (Gladwell and Coote, 2002; Ives et al., 2016; Kruse et al., 2016; Nakamura et al., 2022). In a recent study, we successfully isolated a mechanoreflex-mediated pressor response using static passive stretching (SPS) in the forearm (Nakamura et al., 2023). Thus, it is possible to isolate the effect of the mechanoreflex on the central BP using this recently developed method. Considering previous studies, the metaboreflex mainly contributes the elevation of central BP whereas the mechanoreflex contributes it. It is important to investigate the effect of mechanoreflex on central BP to reveal the mechanism of central BP regulation during exercise.

An increase in the central systolic BP (SBP) associated with the metaboreflex was attributed to the elevation of P2 on P1 (Kalfon et al., 2015). The mechanoreflex may induce different shape changes in the central BP waveform than the metaboreflex, because the mechanoreflex mainly increases stroke volume (SV) (Venturelli et al., 2019; Zambolin et al., 2022), whereas the metaboreflex mainly augments peripheral vasoconstriction (Ichinose et al., 2017). If both the metaboreflex and mechanoreflex increase load of central artery, how they place a load on central artery may be different. Evaluating changes in the shape of the central BP waveform can uncover the difference. No data, to our knowledge, have compared the alteration in the shape of the central BP waveform between the mechanoreflex and metaboreflex.

Thus, the purpose of the present study was to investigate the effect of muscle mechanoreflex on central BP. We hypothesized that the mechanoreflex increases central BP, and mechanoreflex and metaboreflex changes the central BP waveform in different manners.

## 2 Materials and methods

### 2.1 Study design

To achieve the purpose of the present study, we assessed carotid BP (cBP) during SPS for isolation of the mechanoreflex and compared the shape changes of the cBP waveform during the mechanoreflex and metaboreflex. All participants provided written informed consent before the start of the study. The purpose, procedures, and risks involved in this study were reviewed and approved by the Human Research Committee of Waseda University (approval No. 2021–392). The study was conducted in accordance with the principles of the Declaration of Helsinki (1975). Participants were asked to avoid food and caffeine intake for at least 12 h and alcohol intake and intensive exercise for at least 24 h of the experiment. The absence of muscle pain, such as delayed-onset muscle soreness, was confirmed by a questionnaire before measurements were performed.

### 2.2 Participants

We recruited 18 healthy males (age, 26 ± 3 years; height, 172.7 ± 6.1 cm; body mass, 68.4 ± 13.2 kg; body mass index [BMI], 22.8 ± 3.7 kg/m^2^; body fat, 18.4% ± 5.3%; maximal voluntary contraction [MVC] of handgrip, 415.5 ± 93.1 N; means ± standard deviation [SD]) in this study. The participants eligibility criteria were normotensive, non-smoker, free of cardiovascular disease or diabetes, according to their medical histories.

### 2.3 Protocol

After measurement of body composition, all participants were put a three-lead electrocardiogram (BSM-2401, Nihon Kohden, Tokyo, Japan), finger photoplethysmography (Finometer MIDI, Finapres Medical Systems, Amsterdam, Netherlands) and oscillometric (Form PWV/ABI BP-203RPEⅡ and TU-100, Colin Medical, Komaki, Japan) equipped with the applanation tonometer probe (CAP-350, Colin Medical, Komaki, Japan) device in supine position. They then took a 15-min rest in the supine position. After that, all participants performed SPS of the right forearm for 60 s. During a preliminary trial, the wrist joint of the subject was stretched by an experimenter to determine the pain threshold and the adequate target magnitude of the stretch. They also performed 120-s isometric handgrip (IHG) at 30% maximal voluntary contraction (MVC), underwent 180-s post-exercise ischemia (PEI), and took a 15-min recovery in the supine position. Maximal voluntary contraction (MVC) of right handgrip were measured in the supine position before IHG. The cBP waveform was obtained from the right common carotid artery as the central BP waveform during these procedures except for the determination of adequate target magnitude of the stretch and measurement of MVC.

We evaluated the P1 and P2 from the cBP waveform. We also assessed the relative change (Δ) from rest in SPS and PEI in stroke volume (SV), total peripheral resistance (TPR), carotid SBP (cSBP), P1 and augmentation pressure (AP).

#### 2.3.1 Static passive muscle stretching for isolation of muscle mechanoreflex

**FIGURE 1:**
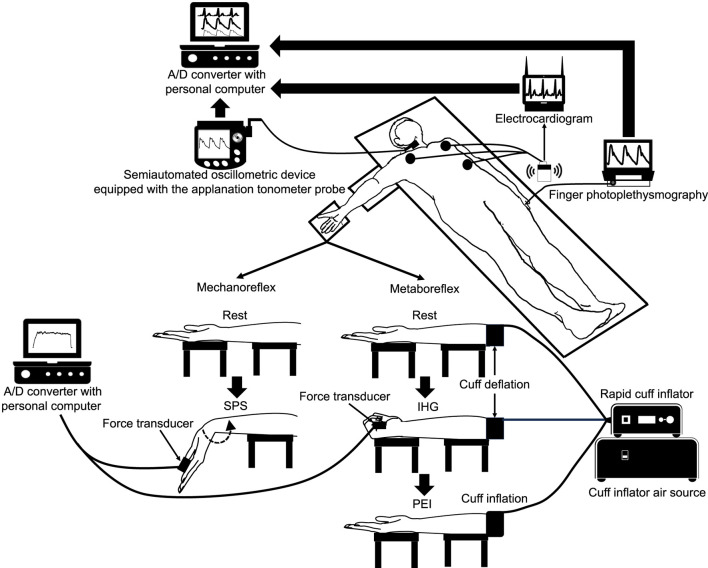
Schematic diagram of the experimental setup and the position of the hand during rest, static passive stretching, isometric handgrip and post-exercise ischemia. A/D, analogue/digital; IHG, isometric handgrip; PEI, post-exercise ischemia; SPS, static passive stretching.

SPS of the forearm was applied to isolate muscle mechanoreflex as described in our previous study (Nakamura et al., 2023). After a 15-min rest period in the supine position with a neutral (0°) wrist joint angle, all participants completed SPS of the forearm for 60 s (Figure 1). The values of force were displayed to provide visual feedback to the experimenter. To minimize the startle reflex to the passive stretching, subjects were made aware that passive stretching would take place in 1 min before starting SPS. All participants were instructed to remain relaxed to prevent voluntary resistance during SPS. During a preliminary trial, the wrist joint of the subject was stretched by an experimenter to determine the pain threshold and the adequate target magnitude of the stretch. The wrist joint was passively stretched by the experimenter at just under the pain threshold during the SPS (Nakamura et al., 2023). The wrist angle from just beside the stretched hand was recorded using a camera (iPhone 8; Apple, Cupertino, CA) located just beside the right hand, and the recordings were analyzed using ImageJ software (National Institutes of Health, Bethesda, MD). After the end of SPS, the 60 s recovery data were also recorded. All cardiovascular data were calculated at rest (averaged over the last 15 s before starting SPS), during SPS at 30 s (averaged at 15–30 s) and 60 s (45–60 s), and during recovery at 60 s (45–60 s).

#### 2.3.2 Post-exercise ischemia for the isolation of muscle metaboreflex

The participants rested quietly for 15 min in the supine position. They then performed a 120-s isometric handgrip (IHG) at 30%MVC. The force output was displayed on a screen to provide visual feedback to participants. All participants were instructed to maintain normal breathing to prevent Valsalva maneuver during IHG. Five seconds before termination of the contraction, the cuff pre-attached around the participant’s upper arm was rapidly inflated to 200 mmHg using a rapid cuff inflator (E20 [rapid cuff inflator] and AG101 [cuff inflator air source], D.E. Hokanson Inc., WA, United States). The cuff remained inflated for an additional 180 s as post-exercise ischemia (PEI) to isolate muscle metaboreflex after the IHG (Figure 1). Following cuff deflation, a 180-s recovery period was conducted. All cardiovascular data were calculated at rest (averaged over the last 15 s before starting IHG), IHG at 60 s (averaged 45–60 s [IHG1]), and 120 s (90–120 s [IHG2]); PEI at 60 s (averaged over the last 45–60 s [PEI1]), 120 s (90–120 s [PEI2]), and 180 s (165–180 s); and recovery at 60 s (45–60 s), 120 s (90–120 s), and 180 s (165–180 s).

These measurements were performed same day under comfortable laboratory conditions between 0900 and 1200.

### 2.4 Body composition

Body composition was measured using bioelectrical impedance analysis (InBody 720; InBody Japan Inc., Tokyo, Japan) with the participants in the upright position.

### 2.5 Brachial blood pressure and cardiac variables

The heart rate (HR) and beat-to-beat peripheral BP waveforms were monitored using a three-lead electrocardiogram (BSM-2401, Nihon Kohden, Tokyo, Japan) and finger photoplethysmography (Finometer MIDI, Finapres Medical Systems, Amsterdam, Netherlands), respectively. The probe of the latter was attached to the middle finger of the subject’s left hand. The BP value obtained from finger was automatically reconstructed as brachial BP value (Beatscope, version 1.1, Finapres Medical Systems, Amsterdam, Netherlands) (Figure 1). SV was assessed based on the obtained brachial BP waveform using the model flow method (Wesseling et al., 1993), which incorporates age, height, and body mass, and simulates aortic flow waveforms from an arterial pressure signal using a nonlinear three-element model of the aortic input impedance (Beatscope, version 1.1, Finapres Medical Systems, Amsterdam, Netherlands). Cardiac output (CO) and TPR were then calculated as SV × HR and mean arterial pressure (MAP)/CO, respectively. The individual relative changes from rest were used for statistical analysis of the hemodynamic variables.

### 2.6 Carotid blood pressure waveform

cBP waveform was obtained in the right common carotid artery. The obtained pressure waveforms were converted from a semiautomated oscillometric device (Form PWV/ABI BP-203RPEⅡ and TU-100, Colin Medical, Komaki, Japan) equipped with the applanation tonometer probe (CAP-350, Colin Medical, Komaki, Japan) at a sampling rate of 1,000 Hz through an analog/digital converter (PowerLab/16SP, AD Instruments, NSW, Australia) and recorded in a device connected to a personal computer (Macbook Pro, Apple, CA, United States) (Figure 1). Then, the obtained data were analyzed using an analysis software (LabChart8, AD Instruments, NSW, Australia). The cBP was calibrated by equating the carotid diastolic BP (DBP) and MAP to the brachial BP value as previously described (Armentano et al., 1995). The coefficient of variation for the carotid systolic BP (cSBP) was 5.0% ± 3.2% within the same participant.

**FIGURE 2:**
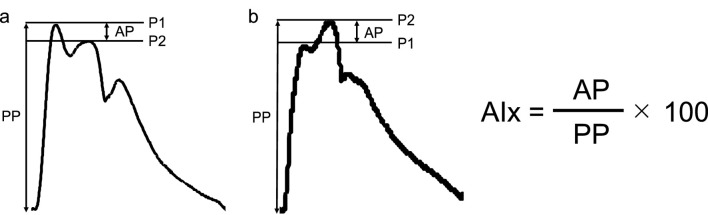
Example of a carotid blood pressure waveform separated into its components in negative **(A)** and positive **(B)** augmentation pressure. AIx augmentation index, AP, augmentation pressure; PP, pulse pressure; P1, first systolic peak; P2, second systolic peak.

The cBP waveform was analyzed as shown in Figure 2. We evaluated P1 and P2, and then calculated AP as the difference between P1 and P2. Negative (Figure 2A) and positive (Figure 2B) AP were defined as those under and over P2, respectively. The AP was divided by the percentage of the overall pulse pressure to calculate the augmentation index (AIx). The coefficient of variation for the P1, P2 was 3.9% ± 4.5%, 3.1% ± 2.2% within the same participant, respectively.

### 2.7 Force

The force generated by SPS was recorded using a force transducer (MLT004/ST; ADInstruments, NSW, Australia). The force signals were converted from the force transducer at a sampling rate of 1,000 Hz by an A/D converter (PowerLab/16SP, ADInstruments, NSW, Australia) and recorded to a personal computer (DynaBook, Toshiba, Tokyo, Japan) (Figure 1). The force data were calculated during SPS at 30 s (averaged at 15–30 s) and 60 s (45–60 s). Data were analyzed using LabChart software (LabChart8, ADInstruments, NSW, Australia).

### 2.8 Maximal voluntary contraction

The MVC of handgrip was measured with a force transducer (MLT004/ST; ADInstruments, NSW, Australia) in the arm extended with supine position (Figure 1). The participants then gripped the force transducer as strongly as possible for 3 s without pressing the instrument against their body or bending at the elbow. The MVC were defined as the averages of two trials for the right arm.

### 2.9 Statistical analysis

*A priori* statistical power analysis was performed to determine the sample size needed for the present study using G*Power 3.1.9.6. We calculated the sample size to investigate the effect of mechanoreflex on central BP. We determined that a sample size of 10 was needed to achieve a statistical power (1 − *β*) of more than 0.80, which was required to reject the null hypothesis, with a large standardized effect size (*f* = 0.40) and an error probability of 0.05 (*α*), using a repeated measure one-way analysis of variance (ANOVA) (number of measurements = 4). Assuming there will be some exclusion, we recruited 16 participants.

All data are expressed as the mean ± standard deviation (SD). Statistical analyses were performed using IBM SPSS Statistics for Windows version 27.0 (IBM Corp., Armonk, NY, United States). The effect of time was examined using a one-way repeated-measures ANOVA. We calculated the relative change (Δ) from rest in SPS and PEI in SV, TPR, cSBP, P1 and AP. The changes in cSBP, P1 and AP during SPS and PEI were also examined using the paired Student’s t*-*test. The Shapiro–Wilk test was performed to determine the normality of data. Pearson’s correlations were used to assess the relationships these Δ. The level of significance for all comparisons was set at *p* < 0.05.

## 3 Results

**FIGURE 3:**
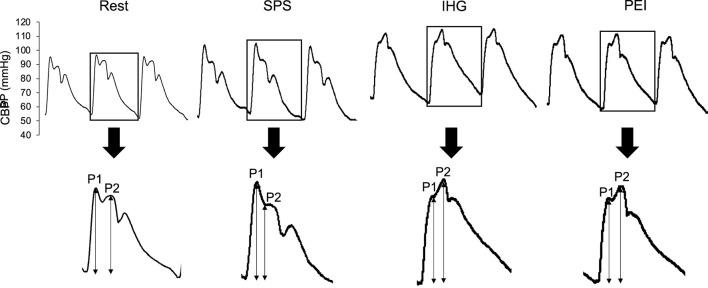
Typical response of central blood pressure waveform during rest, static passive stretching, isometric handgrip and post-exercise ischemia. BP, blood pressure; IHG, isometric handgrip; PEI, post-exercise ischemia; P1, first systolic peak; P2, second systolic peak; SPS, static passive stretching.

Data of six participants were excluded from the analysis since central BP waveform was not successfully obtained. Then the results were based on the data of the remaining 12 participants (age, 26 ± 2 years; height, 171.1 ± 5.2 cm; body mass, 63.3 ± 10.3 kg; BMI, 21.5 ± 2.6 kg/m^2^; body fat, 16.7% ± 3.9%; MVC, 39.5 kg). Figure 3 shows typical response of cBP waveform during rest, SPS, IH and PEI.

### 3.1 Response of the brachial and carotid blood pressures and cardiac variables during static passive stretch

Table 1 summarizes the responses of the brachial BP and cardiac variables during SPS. The SV significantly increased at 30 and 60 s during SPS compared to that at rest (*p* < 0.05). Brachial SBP (bSBP), DBP, MAP, SV, and TPR were significantly elevated at 60s during SPS compared to those at rest (*p* < 0.05). There were no significant differences in brachial pulse pressure (bPP), HR and CO from rest during SPS. There was a significant difference in the SV and force at 30 and 60 s during SPS (*p* < 0.05).

**TABLE 1:** Response of brachial hemodynamics and cardiac variables during static passive stretching.

	Rest	SPS30	SPS60	RE
bSBP mmHg	103 ± 7	108 ± 8	110 ± 8*	102 ± 7
DBP mmHg	59 ± 9	62 ± 11	64 ± 10*	59 ± 8
MAP mmHg	75 ± 9	79 ± 11	82 ± 10*	74 ± 8
bPP mmHg	46 ± 6	46 ± 8	46 ± 10	45 ± 7
HR bpm	59 ± 8	57 ± 7	54 ± 7	56 ± 8
SV mL	84 ± 8	94 ± 12*	97 ± 13*	93 ± 14
CO L/min	5.0 ± 0.9	5.3 ± 0.9	5.2 ± 1.0	5.2 ± 1.1
TPR mmHg/L/min	15.3 ± 2.4	15.0 ± 2.3	16.1 ± 2.4*	14.5 ± 2.4
Force N	0.0 ± 0.0	57.0 ± 19.9*	56.8 ± 21.1*	0.0 ± 0.0
Wrist joint angle °		86.2 ± 11.8	88.7 ± 9.3	

Values are presented as mean ± standard deviation. bSBP, brachial systolic blood pressure; DBP, diastolic blood pressure; MAP, mean arterial pressure; bPP, brachial pulse pressure; HR, heart rate; SV, stroke volume; CO, cardiac output; TPR, total peripheral resistance; bPP, brachial pulse pressure. *Significantly different (*p* < 0.05) from compared with rest.

**FIGURE 4:**
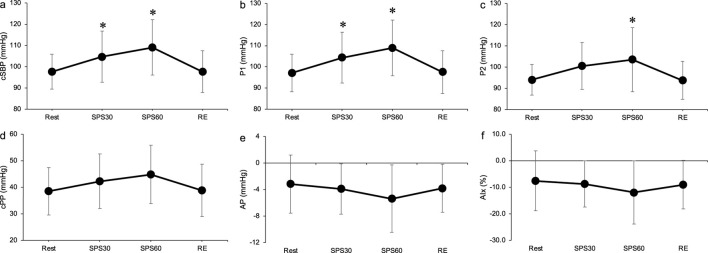
Time course of carotid systolic blood pressure **(A)**, P1 **(B)**, P2 **(C)**, carotid pulse pressure **(D)**, augmentation pressure **(E)** and augmentation index **(F)** during static passive stretching. Values are presented as mean ± standard deviation. cSBP, carotid systolic blood pressure; P1, first peak; P2, second peak; cPP, carotid pulse pressure; AP, augmentation pressure; AIx, augmentation index; SPS, static passive stretching; RE, recovery. *Significantly different (*p* < 0.05) compared with rest.

Figure 4 shows the time course of the cBP during SPS. cSBP and P1 were significantly elevated at 30 and 60 s during SPS from rest (*p* < 0.05, Figures 4A, B). P2 significantly increased at 60 s during SPS compared to rest (*p* < 0.05, Figure 4C). There were no significant differences in the other cBP variables.

### 3.2 Response of the brachial and central blood pressures and cardiac variables during isometric handgrip and post-exercise ischemia

Table 2 summarizes the brachial BP and cardiac variables during IHG and PEI. The bSBP, DBP, and MAP were significantly elevated at all points in the IHG and PEI from rest (*p* < 0.05). The TPR significantly increased during IHG and PEI compared to the rest, without IHG1 (*p* < 0.05). bPP showed significant augmentation only in the PEI3 compared to rest (*p* < 0.05). HR and CO were significantly elevated only in the IHG group compared to rest (*p* < 0.05). SV increased significantly only in PEI (*p* < 0.05).

**TABLE 2:** Response of brachial hemodynamics and cardiac variables during isometric handgrip and post-exercise ischemia.

	Rest	IHG1	IHG2	PEI1	PEI2	PEI3	RE1	RE2	RE3
bSBP mmHg	107 ± 7	123 ± 9*	134 ± 14*	125 ± 10*	125 ± 10*	127 ± 10*	116 ± 8	114 ± 7	114 ± 6
DBP mmHg	64 ± 6	73 ± 8*	81 ± 9*	72 ± 8*	72 ± 7*	72 ± 7*	65 ± 7	65 ± 6	65 ± 5
MAP mmHg	81 ± 5	90 ± 9*	100 ± 12*	92 ± 7*	92 ± 7*	92 ± 7*	83 ± 6	82 ± 6	83 ± 5
bPP mmHg	43 ± 7	49 ± 6	53 ± 7	53 ± 7	53 ± 7	54 ± 8*	51 ± 6	49 ± 7	49 ± 5
HR bpm	59 ± 6	66 ± 8*	68 ± 9*	53 ± 7	55 ± 8	56 ± 7	57 ± 7	58 ± 8	57 ± 8
SV mL	94 ± 12	94 ± 15	94 ± 13	106 ± 21*	106 ± 19*	108 ± 19*	104 ± 18	99 ± 16	101 ± 15
CO L/min	5.5 ± 0.8	6.2 ± 1.1*	6.4 ± 1.1*	5.6 ± 0.9	5.7 ± 0.8	5.9 ± 0.9	5.9 ± 0.8	5.7 ± 0.7	5.7 ± 0.9
TPR mmHg/L/min	15.0 ± 2.6	15.0 ± 2.9	16.0 ± 2.3*	16.7 ± 2.7*	16.4 ± 2.3*	15.7 ± 2.2*	14.4 ± 1.8	14.6 ± 1.8	14.8 ± 2.3
Force output kg	0.0 ± 0.0	11.5 ± 2.5*	11.5 ± 2.3*	0.0 ± 0.0	0.0 ± 0.0	0.0 ± 0.0	0.0 ± 0.0	0.0 ± 0.0	0.0 ± 0.0
%MVC %	0.0 ± 0.0	29.2 ± 1.0*	29.0 ± 2.2*	0.0 ± 0.0	0.0 ± 0.0	0.0 ± 0.0	0.0 ± 0.0	0.0 ± 0.0	0.0 ± 0.0

Values are presented as mean ± standard deviation. bSBP, brachial systolic blood pressure; DBP, diastolic blood pressure; MAP, mean arterial pressure; bPP, brachial pulse pressure; HR, heart rate; SV, stroke volume; CO, cardiac output; TPR, total peripheral resistance; MVC, maximal voluntary contraction. *Significantly different (*p* < 0.05) fromcompared withe rest.

**FIGURE 5:**
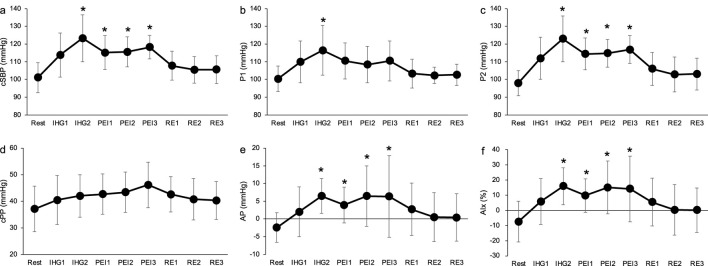
Time course of carotid systolic blood pressure **(A)**, P1 **(B)**, P2 **(C)**, carotid pulse pressure **(D)**, augmentation pressure **(E)** and augmentation index **(F)** during isometric handgrip and post-exercise ischemia. Values are presented as mean ± standard deviation. cSBP, carotid systolic blood pressure; P1, first systolic peak; P2, second systolic peak; cPP, carotid pulse pressure; AP, augmentation pressure; AIx, augmentation index; IHG, isometric handgrip; PEI, post-exercise ischemia; RE, recovery. *Significantly different (*p* < 0.05) compared with rest.

Figure 5 shows the time course of the cBP variables during IHG and PEI. cSBP, P2, AP and AIx were significantly elevated in IHG2, PEI1, PEI2 and PEI3, compared to the rest (*p* < 0.05, Figures 5A, C, E, F). There was a significant difference in P1 in IHG2 at rest (*p* < 0.05, Figure 5B). There was no significant difference in the cPP at rest during IHG and PEI (Figure 5D). Changes in the carotid blood pressure during static passive stretching and post-exercise ischemia.

**FIGURE 6:**
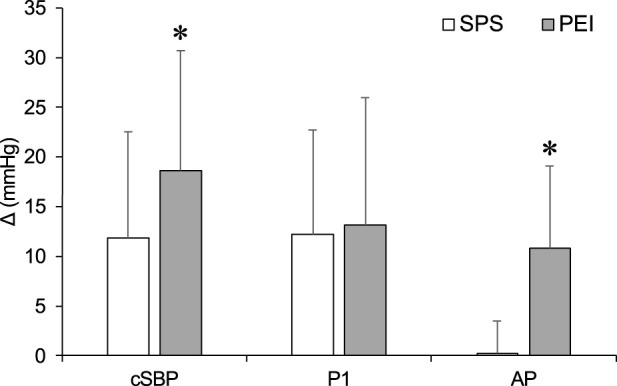
Changes in carotid systolic blood pressure, P1 and augmentation pressure during static passive stretching and post-exercise ischemia. Values are presented as mean ± standard deviation. cSBP, carotid systolic blood pressure; P1, first systolic peak; AP, augmentation pressure; SPS, static passive stretching; PEI, post-exercise ischemia. *Significantly different (*p* < 0.05) compared with SPS.

Figure 6 shows changes in cSBP, P1, and AP during SPS and PEI. The ΔcSBP and ΔAP were significantly higher in PEI than in SPS (*p* < 0.05, Figure 6). There were no significant differences in ΔP1 between SPS and PEI.

### 3.3 Relationship between changes in stroke volume or total peripheral resistance and first systolic peak or augmentation pressure

Table 3 shows the association between ΔSV or ΔTPR or ΔMAP and ΔP1 or ΔAP. A significant positive correlation was found between ΔSVΔP1 and ΔSV or ΔMAP ΔP1 during SPS (ΔP1 and ΔSV: *r* = 0.68, *p* < 0.05; ΔP1 and ΔMAP: *r* = 0.90, *p* < 0.05). During PEI, ΔTPR and ΔMAP were significantly correlated with ΔAP (ΔAP and ΔTPR: *r* = 0.61, *p* < 0.05; ΔAP and ΔMAP: *r* = 0.62, *p* < 0.05). No significant correlation was found between ΔP2 and ΔcSBP (*r* = 0.49, *p* = 0.11). An elevation in TPR was observed during PEI, and ΔTPR during PEI was related to ΔAP (Table 3).

**TABLE 3:** Association between changes in stroke volume or total peripheral resistance and changes in first systolic peak or augmentation pressure during static passive stretching.

	ΔSV		ΔTPR		ΔMAP	
	*R* value	*p*-value	*R* value	*p*-value	*R* value	*p*-value
SPS (n = 12)						
ΔP1	0.68	<0.05	0.43	0.16	0.90	<0.05
ΔAP	0.36	0.25	0.13	0.69	−0.01	0.98
PEI (n = 12)						
ΔP1	0.22	0.49	0.36	0.25	0.62	<0.05
ΔAP	−0.20	0.53	0.61	<0.05	−0.45	0.14

SPS, static passive stretching; PEI, post-exercise ischemia; SV, stroke volume; TPR, total peripheral resistance.

## 4 Discussion

The present study investigated the effect of muscle mechanoreflex on central BP and compared the changes in the shape of the central BP waveform during mechanoreflex and metaboreflex. The major findings of the present study were that SPS increased cSBP with an elevation of P1, whereas PEI increased cSBP with an elevation of P2. SPS did not alter AP, whereas PEI increased it. The ΔP1 during SPS was positively correlated with ΔSV, and ΔAP during PEI was positively correlated with ΔTPR. These results suggest that both the metaboreflex and mechanoreflex increase cSBP through different mechanisms.

We observed the effect of muscle mechanoreflex on central BP. The SPS, isolating the muscle mechanoreflex, increased cSBP and P1 (Figures 4A, B). Previous study has reported that central BP elevated during static exercise (Chirinos et al., 2010). Elevation of cSBP during SPS suggests that the mechanoreflex contributes central BP regulation. A significant positive correlation was also found between ΔSV and ΔP1 during SPS (ΔP1 and ΔSV: *r* = 0.68, *p* < 0.05, Table 3). These results suggest that the cardiac contractile force influences the augmentation of cSBP during the mechanoreflex. Previous studies have reported that P1 is generated by cardiac ejection, such as SV (Nichols, 2005; Cecelja and Chowienczyk, 2012). The increase in P1 during SPS was reflected by an increase in SV due to the mechanoreflex. Consistent with other studies, the present study demonstrated that passive muscle stretch causes a muscle mechanoreflex-mediated SV increase in humans (Venturelli et al., 2019; Zambolin et al., 2022). In addition, previous animal studies have suggested that the stimulation of mechanosensitive receptors increases cardiac sympathetic nerve activity (Matsukawa et al., 1994). We can only speculate that the increase in SV during SPS in the present study was mainly a consequence of the elevation of cardiac sympathetic nerve activity associated with mechanoreflex, and that SV contributes to the elevation of P1 during SPS as we did not measure cardiac sympathetic nerve activity.

Different changes were observed in the shape of the central BP wave during metaboreflex and metaboreflex, though both the metaboreflex and mechanoreflex increased cSBP (Figures 4A, 5A), The SPS, isolating the mechanoreflex, increased P1 and maintained negative AP (Figures 4A, B) whereas the PEI, isolating the metaboreflex, increased P2 and altered positive AP (Figures 5A, B). A significant positive correlation was also observed ΔSV and ΔP1 during SPS, and ΔTPR and ΔAP during PEI (Table 3). The changes in the shape of the central BP waveform was different between the mechanoreflex and the metaboreflex.

The different waveform must be explained by different effects of metaboreflex and mechanoreflex on P1 and P2 waves. Previous studies have reported that P1 and P2 are influenced by SV and peripheral vasoconstriction, respectively (Nichols, 2005; Cecelja and Chowienczyk, 2012). Alteration of SV with the mechanoreflex was mentioned above. P2 mainly contributes the elevation of central SBP with the metaboreflex (Kalfon et al., 2015). The metaboreflex increases vasoconstriction (Ichinose et al., 2006; 2017; Fisher et al., 2008; 2015; Crisafulli et al., 2015; Ogoh et al., 2019), and peripheral vasoconstriction induces the augmentation of P2 and AP (Cecelja and Chowienczyk, 2012). These relates to an increase in muscle sympathetic activity (Scherrer et al., 1990). Metaboreflex-induced sympathoexcitation increases P2 and AP, resulting in elevated cSBP. Thus both the metaboreflex and mechanoreflex increase cSBP, whereas the shape changes in the central BP waveform are different.

Peripheral BP such as brachial BP is not always a reliable assessment of ascending central BP and thus can underestimate central artery and the left ventricular workload (Westerhof and O’Rourke, 1995). A study suggested that P2 response to static handgrip is stronger predictor of left ventricular hypertrophy than P1 (Chirinos et al., 2010). Present study has suggested that the elevation of P2 is mainly induced the metaboreflex. Thus, assessing the waveform during metaboreflex induced by PEI may be useful predictor of the left ventricular workload during static exercise and potentially left ventricular hypertrophy. Still the effect of increase in P2 with metaboreflex on left ventricle is unclear. The contribution of P2 with the metaboreflex on left ventricle should be investigated in future studies.

There were slightly different time courses of mechanoreflex-induced pressor response between the present study and previous studies. During SPS, both bSBP and P2 were significantly elevated at SPS60 compared to rest, no significant difference was observed at SPS30. The muscle mechanoreflex also had an important role in the modulation of BP at the onset of exercise (∼30 s) during previous studies (Gladwell and Coote, 2002). The mechanisms of the different results should be investigated in future studies.

The elevation of P2, AP, and AIx with PEI suggests augmentation of reflected waves from the peripheral arteries to the heart. The major reflection sites are located in the arterial tree of the lower limbs at rest (Westerhof et al., 2008; Sugawara et al., 2010). The PEI of the arm was reasonably considered to augment the reflected wave in the present study because it increased the leg vascular resistance in a previous study (Ichinose et al., 2007), although we did not measure it. In the present study, we could not separate the reflected wave from the metaboreflex and/or vasoconstriction directly using arm occlusion-induced vasocontraction PEI. Arm occlusion may have a marginal effect on the reflected wave, because the arm volume is much smaller than the lower limb volume. Future studies are needed to clarify the location of the major reflection site during PEI of the arm.

This study had other limitations. First, only male participants were recruited for the present study. Previous studies have reported sex-specific differences in the mechanoreflex (Ives et al., 2013; Fouladi et al., 2019) and metaboreflex responses (Lee et al., 2021). Second, only healthy young participants were included. Older males have greater absolute central BP during light-to moderate-intensity exercise than younger ones (Casey et al., 2008). Future studies should examine the effects of sex and age on the EPR-mediated central BP response to exercise. Third, this study was concerned with the validity of central BP estimation using brachial BP values reconstructed from finger BP values. Finger arterial waveform-derived pulse wave analysis measurements have been suggested to have a high interchange ability for estimating central BP values (Sugawara et al., 2015). Forth, we did not evaluate muscle activation during SPS. It is important to conform muscle inactivity during SPS. Fifth, we can not identify which ion channels are activated by SPS. We should investigate the molecules mechanisms of pressor response during SPS. Finally, we did not measure central arterial stiffness, which is associated with central BP (Pietschner et al., 2023). Increased central arterial stiffness promotes a faster return of reflected waves from peripheral arterial sites to the aorta, increasing the central BP (Nichols, 2005).

### 4.1 Conclusions

The results of the present study suggest that the mechanoreflex contributes to an increased central BP. It also suggests that the shape change of the central BP waveform with the mechanoreflex is different from that of the metaboreflex, which may primarily deform P1 and P2. Further studies are needed to investigate the central BP regulation during exercise based on the present study since previous studies have shown that the increases in central SBP response to exercise are different between peripheral and central BP (Rowell et al., 1968).

## Data Availability

The raw data supporting the conclusions of this article will be made available by the authors, without undue reservation.
